# Neuroblastoma-derived hypoxic extracellular vesicles promote metastatic dissemination in a zebrafish model

**DOI:** 10.1371/journal.pone.0316103

**Published:** 2024-12-23

**Authors:** Anna Fietta, Pina Fusco, Giuseppe Germano, Sara Micheli, Marco Sorgato, Giovanni Lucchetta, Elisa Cimetta

**Affiliations:** 1 Department of Biomedical Sciences (DSB), University of Padua, Padova, Italy; 2 Fondazione Istituto di Ricerca Pediatrica Città della Speranza (IRP), Padova, Italy; 3 Department of Industrial Engineering (DII), University of Padua, Padova, Italy; Hirosaki University Graduate School of Medicine, JAPAN

## Abstract

The zebrafish (*Danio rerio*) is a valuable model organism for studying human biology due to its easy genetic manipulation and small size. It is optically transparent and shares genetic similarities with humans, making it ideal for studying developmental processes, diseases, and drug screening via imaging-based approaches. Solid malignant tumors often contain hypoxic areas that stimulate the release of extracellular vesicles (EVs), lipid-bound structures released by cells into the extracellular space, that facilitate short- and long-range intercellular communication and metastatization. Here we investigate the effects of EVs derived from neuroblastoma (NB), a pediatric solid tumor, on metastatic niche formation using the zebrafish as an *in vivo* model. Intravascular injection in zebrafish embryos allows a non-invasive visualization of EVs dispersion, uptake, and interactions with host cells. To improve repeatability of our results and ease the injection steps, we used an agarose device replica molded from a custom designed micromilled aluminum mold. We first demonstrated that EVs released under hypoxic conditions promote angiogenesis and are more easily internalized by endothelial cells than those purified from normoxic cells. We also showed that injection of with hypoxic EVs increased macrophages mobilization. We then focused on the caudal hematopoietic tissue (CHT) region of the embryo as a potential metastatic site. After hypoxic EVs injection, we highlighted changes in the expression of *mmp-9* and *cxcl8b* genes. Furthermore, we investigated the ability of NB-derived EVs to prime a metastatic niche by a two-step injection of EVs first, followed by NB cells. Interestingly, we found that embryos injected with hypoxic EVs had more proliferating NB cells than those injected with normoxic EVs. Our findings suggest that EVs released by hypoxic NB cells alter the behavior of recipient cells in the zebrafish embryo and promote metastatic outgrowth. In addition, we demonstrated the ability of the zebrafish embryo to be a suitable model for studying the interactions between EVs and recipient cells in the metastatic process.

## Introduction

Metastasis is the major contributor to cancer mortality, accounting for 90% of all cancer-related deaths. This complex process involves the mobilization of tumor cells from the primary site, their intravasation into the bloodstream, subsequent arrest, and extravasation at the target site, while favoring angiogenesis and proliferation at distant locations within the body [1]. Among the several active players in this process, extracellular vesicles (EVs), cell-secreted membrane-enclosed particles, emerged over the past decade for their ability to facilitate short- and long-range communication between various cell types at different metastatic stages [2]. EVs population is extremely heterogenous and ranges in size from 30 to 10,000 nm in diameter [3]. EVs contain various biomolecules derived from the donor cells, including proteins, nucleic acids, and lipids, and can deliver this molecular information over long distances to recipient cells or tissues within the body. Tumor progression can be promoted by tumor cell derived EVs via induction of cell migration and invasion, stimulation of tumor angiogenesis, and impairment of tumor immunity [4, 5].

Neuroblastoma (NB) is a solid pediatric tumor of neural crest origin [6] characterized by the amplification of the MYC family member *MYCN* oncogene in ∼25% of cases, which correlates with high-risk disease and poor prognosis. Amplification of *MYCN* remains the best-characterized genetic marker of risk in NB. Adrenal medulla, paraspinal ganglia and sympathetic nervous system are common primary NB tumor sites whereas bone, bone marrow and lymph nodes are mainly metastatic sites [7, 8].

The zebrafish (*Danio rerio*) is a powerful *in vivo* model showing a high degree of physiological and genetic similarity to mammals. In its simplicity, it can be used to mimic the complex tumor microenvironment to study human cancer biology [9]. Zebrafish xenotransplantation enables to study cancer progression, neo-angiogenesis, drug response and resistance, as well as metastasis [10, 11], with fluorescent-stained tumor cells easily tracked in the optically transparent embryos allowing live monitoring of the process of metastatization. Zebrafish has been used in numerous studies as a model for NB metastasis [12, 13], but a study investigating the effect of NB derived EVs on metastatic dissemination is still lacking. We here investigated the role of EVs released by NB cells in metastatic progression as a function of the oxygenation level of the parent cells. In NB, evidence point towards a role of hypoxia in tumor initiation during development, tumor cell differentiation, survival, and metastatic spreading [14]. However, the heterogeneous nature of NB, its developmental origin, and the lack of suitable experimental models have hampered a clear understanding of the effect of hypoxia in its progression and the molecular mechanisms implicated [15]. Here, we injected fluorescently labeled NB-derived EVs into zebrafish larvae at 2 days post fertilization (dpf) and investigated their contribution to tumor progression and metastatic colonization. An agarose molded device eased the injection steps and increased repeatability of our results. We report that hypoxic NB-derived EVs enhance angiogenesis and macrophages mobilization and modify the expression of *mmp-9* and *cxcl8b*, genes involved in metastatic progression. Moreover, a two-steps injection of EVs and NB cells allowed us to mimic the metastatic colonization process, suggesting that hypoxic EVs promote metastatic outgrowth compared to their normoxic counterpart. Taken together, our data demonstrate that zebrafish larvae xenotransplants are promising *in vivo* models to investigate the role of EVs-dependent tumorigenesis and metastatic dissemination and show a potential use of EVs as a therapeutic target for NB metastasis.

## Materials and methods

### Cell culture

SK-N-DZ (ATCC, CRL 2149) and IMR32 (kindly provided by Dr G.P. Tonini) cell lines were cultured in DMEM supplemented with 10% FBS (ATCC), 100 U/mL penicillin/streptomycin (Life Technologies) and 1% MEM NEAA (Gibco) at 37°C in 5% CO_2_. Mesenchymal Stem Cells (MSCs) (ATCC, PCS-500-030) were cultured in Mesenchymal Stem Cell Basal Medium (ATCC) supplemented with 125 pg/mL rh FGF-b, 15 ng/mL rh IGF-1, 7% FBS, 2.4 mM L-alanyl-L-glutamine and 0.5% penicillin/streptomycin at 37°C, 5% CO_2_ (studies involving MSCs are reported as Supplementary Information). For hypoxic studies, cells were maintained at 37°C with 5% CO_2_ and 1.5% O_2_ in Whitley H35 Hypoxystation (Don Whitley Scientific).

### Zebrafish

Fish handling procedures and care maintenance were accomplished in accordance with EU Directive 2010/63/EU for animal experiments. All experiments were conducted in non-feeding zebrafish embryos and their protocols were approved by the local OPBA (Organismo Preposto al Benessere Animale) of Fondazione Istituto di Ricerca Pediatrica Città della Speranza (Authorization Number: N02ZFU5). Adult *Casper* (*Danio rerio*) [16] and the transgenic zebrafish lines *Tg(fli1*:*EGFP)* [17], *Tg(mpeg1*:*mCherry)* [18] and *Tg(gata1*:*dsRed)* [19], were maintained using established temperature and light cycle conditions as previously described [20]. Fertilized eggs were obtained from natural spawning. Embryos were kept at 28.5°C in methylene blue-containing water (E3 medium) until injection. For EVs transplantation experiments, 2 dpf embryos were dechorionated and anaesthetized in 0.3× Danieau’s solution containing Phenythiourea (PTU, Sigma-Aldrich), excluded for the Casper embryos, and 0.04 mg/ml Tricaine (Sigma-Aldrich) before injection.

### EVs isolation and labeling

EVs were isolated from conditioned media (CM) by ultrafiltration using 100-kDa MWCO Spin-X 20 mL Concentrator (Corning) following the manufacturer’s instructions. To generate CM, 4.5x10^6^ cells were cultured in complete growth medium for 48 hours (80% confluency) and maintained in normoxic (20% O_2_) or hypoxic (1.5% O_2_) condition. Media was then replaced with DMEM supplemented with 100 U/mL penicillin/streptomycin (Life Technologies) and 1% MEM NEAA (Gibco) without FBS for 48 hours. CM was harvested, centrifuged to remove cellular debris (2000g for 5 min), and filtered by 0.22 μm syringe filters (Millipore). 20 mL of CM were then loaded into the concentrator tube and centrifuged at 3000g for 30 minutes at room temperature. This last step was repeated adding 15 mL of PBS 1x and centrifuged at 3000g for 15 minutes at room temperature. EVs derived from SK-N-DZ and IMR32 cells cultured under normoxic (DZN^EVs^ and IMRN^EVs^ respectively) or hypoxic (DZH^EVs^ and IMRH^EVs^ respectively) conditions, were collected and either used fresh or stored at -80°C. The total amount of proteins in EVs was quantified using Pierce BCA Protein Assays (ThermoFisher). EVs were labeled with the fluorescent Vybrant DiO Cell-Labeling Solution (ThermoFisher) or Vybrant DiI Cell-Labeling Solution (ThermoFisher) as recommended by the manufacturer for *in vivo* imaging of *Tg(gata1*:*dsRed)* and *Tg*(*fli1*:*EGFP*) embryos and the evaluation of EVs internalization. Briefly, 100 μg/mL of isolated EVs were resuspended in 1 mL of PBS 1X mixed with 5 μL of Cell-Labeling Solution for 30 minutes at 37°C. Labeled EVs were washed with 13 mL of PBS 1X, loaded into the concentrator tube, centrifuged at 3000g for 15 minutes at room temperature to remove free dye, and immediately injected. For the control group we used 100 nm fluorescent polystyrene beads (Phosphorex Inc.). For angiogenesis assessment using *Tg*(*fli1*:*EGFP*) embryos, macrophages mobilization in *Tg(mpeg1*:*mCherry)* embryos, and RNA extraction using Casper strain embryos, 100 μg/mL of EVs were resuspended in a solution of Fluorescein isothiocyanate–dextran (Sigma-Aldrich) in PBS 1X prior to injection. Control group was injected with dextran solution without EVs.

### EVs characterization

For transmission electron microscopy (TEM) analysis, one drop of purified EVs (~ 25 μL) was deposited on 400 mesh holey film grid. Filter paper (Whatman) was used to remove the excess liquid. EVs were then stained with 1% uranyl acetate for 2 minutes. Samples were observed with a Tecnai G2 (FEI) transmission electron microscope operating at 100 kV equipped with a Veleta CCD camera (Olympus Soft Imaging System) at the BioImaging Facility (Department of Biology, University of Padova). The concentration of EVs was analyzed using Nanosight NS300 (Malvern) according to the manufacturer’s instructions. Briefly, samples were diluted 1:100 in PBS and analyzed under constant flow conditions at 25°C. For each measurement, three consecutive 60s videos were captured with a camera level of 11. Data were analyzed using NTA software with a detection threshold of 5. For western blot analysis, 30 μg of EVs or fresh cell culture medium (considered as CTR), were diluted 1:4 in Cell Extraction Buffer (ThermoFisher), separated by SDS-PAGE using Bolt 4–12% Bis-Tris Plus gels (Invitrogen). Gels were transferred onto nitrocellulose membranes using Power Blotter Select Transfer Stacks and the Power Blotter System (Invitrogen). Membranes were blocked with I-Block reagent (ThermoFisher) and incubated overnight at 4°C with the following primary antibodies: mouse monoclonal anti-calnexin (Santa Cruz); mouse monoclonal anti-CD63 (abcam); mouse monoclonal anti-CD81 (abcam). Membranes were then incubated with peroxidase-conjugated secondary antibody Goat anti-Mouse (G-21040). Images were captured using Westar Hypernova ECL substrate (Cyanagen) and iBright Western Blot Imaging Systems (Invitrogen).

### EmbryoAligner device: Design, production, and characterization

The injection of exogenous substances such as EVs or cells can be invasive for zebrafish embryos. To minimize the manipulation while easing and speeding up embryo transfer processes before and after the injections of EVs or cells, we created a device we named “EmbryoAligner”. The master was fabricated by micro-milling an aluminum block (Kugler MICROMASTER^®^ 5X) based on the chosen design digitalized using 3D-CAD software. The device could then be produced by replica molding from the aluminum master using either Polydimethylsiloxane (PDMS, Sylgard 184 Dow Corning, USA) or agarose using standard techniques [21]. Briefly, PDMS was prepared by mixing the base and the curing agent in a 10:1 w/w ratio, degassed, and cast on the aluminum master. After an additional degassing step, the mold was baked in an oven for 60 minutes at 80°C. Similarly, hot 1.5% agarose was poured over the master and allowed to solidify (approximately 5 minutes). The PDMS and agarose replicas could then easily be peeled from the mold. Optical metrological tests were performed using a 3D optical profiler (Sensofar^®^ S Neox) on both the aluminum master and the PDMS replicas to evaluate the precision of the features. This is important since variations in the heights of the structural elements may compromise the proper alignment and manipulation of the zebrafish embryos. The key measurements are the height of the tail and the main body of the embryo impressed in the mold. PDMS was used for the metrological characterization thanks to its optical properties, while agarose proved ideal for all other experiments.

### Zebrafish EVs transplantation

For EVs transplantation experiments, 2 dpf embryos were dechorionated and anaesthetized in 0.3× Danieau’s solution containing PTU, excluding for the Casper embryos, and 0.04 mg/ml Tricaine. Embryos were then positioned in the EmbryoAligner. A total of 23 nL of labeled EVs or dextran resuspended EVs, were back-loaded into pulled borosilicate glass capillary needles (Narishige PC-10, Drummond Scientific) and injected into the Duct of Cuvier of each embryo using the Drummond Nanoject II microinjector (Drummond Scientific) under a fluorescent stereomicroscope (SMZ1500 Nikon). Upon bloodstream assessment, incorrectly injected embryos were discarded, while other were transferred to E3 medium and either immediately imaged under a confocal microscope or incubated at 34°C until the desired time point. At 5 dpf, zebrafish were sacrificed with 2.4 mM Tricaine. The control group was injected with dextran solution without EVs.

### Confocal imaging and Image-J analysis

Confocal imaging was performed using a Zeiss LSM800 Airyscan. To visualize EVs in the blood circulation, *Tg(gata1*:*dsRed)* and *Tg*(*fli1*:*EGFP*) embryos were imaged immediately after injection. To determine the proportion of EVs internalized by endothelial cells, 6 to 20 Z-stacks were acquired in the caudal plexus of each EVs-injected *Tg(fli1*:*EGFP)* embryo 54 hpf at 25X magnification. We merged stacks into a single image using ImageJ software (National Institutes of Health, MD, United States), measured the colocalization of the green and red signals using the JACoP BIOP plugin, and obtained the Pearson’s correlation coefficient (values ranging from 0 to 1) for each image. To determine the effect of EVs on angiogenesis, we analyzed the increase of sprout area in the sub-intestinal veins (SIVs) of *Tg(fli1*:*EGFP)* embryos [22] 24 hours after EVs injection. 2 to 10 Z-stacks were captured in each zebrafish SIVs at 10X magnification. Using ImageJ software, stacks were merged into a single image and the area was measured after setting a threshold and selecting the SIVs perimeter. To determine the effect of EVs on the immune compartment, we used *Tg(mpeg1*:*mCherry*) embryos, which express fluorescent macrophages. Six hours after EVs injection, embryos were imaged at 25X magnification (10 to 18 Z-stacks). Using ImageJ, stacks were merged into a single image and the area covered by macrophages was measured after setting the proper threshold.

### Tube formation assay

Human Umbilical Vein Endothelial Cells (HUVEC, Lonza) were seeded into a Matrigel Basement Membrane Matrix (354234, Corning) precoated 96-well plate at 2×10^4^ cells/well and cultured in Endothelial Basal Medium, in the presence of EVs (20 μg/mL) or PBS (control). Tube formation was examined using the EVOS FL Cell Imaging System. The angiogenic property was assessed by measuring the total branching point and tube length from five random microscopic fields using Python Angiogenesis analyzer. Results are provided as Supplementary information (Please see S1 Fig).

### RNA extraction and qPCR of CHT tissues

To assess the effects of EVs in the caudal hematopoietic tissue (CHT) 24 hours after EVs injection, 30 Casper strain embryos per condition were placed in a petri dish and anesthetized with 200 mg/L Tricaine to cut CHT sections with a sterile scalpel (Swann-Morton). Sections were collected in 1.5 mL tubes and washed with PBS 1X. Total RNA was extracted using Trizol reagent (Invitrogen), and RNA was quantified using a Nanodrop spectrophotometer (Thermo Fisher Scientific). Isolated RNA was used for cDNA synthesis using the High-Capacity cDNA Reverse Transcription Kit (Applied Biosystem) according to the manufacturer’s instructions. Real-time PCR was performed using Power Up SYBR Green Master Mix (Applied Biosystems) according to the manufacturer’s instructions. Relative mRNA expression for each gene was analyzed by the ddCt method using *β-Actin* as a housekeeping gene. A list of all primers is provided in the S1 Table.

### Two-steps injection, xenotransplantation, imaging, and analysis

*Tg(fli1*:*EGFP)* zebrafish embryos were dechorionated at 48 hpf, anesthetized with 200 mg/L Tricaine and placed on the EmbryoAligner containing a solution of 1.5% agarose in E3 medium. 100 μg/mL of EVs were resuspended in a solution of Fluorescein isothiocyanate–dextran in PBS 1X, loaded into borosilicate glass capillary needles, and 23 nL of suspension was injected into the Duct of Cuvier of each embryo using the Drummond Nanoject II microinjector under the fluorescent stereomicroscope. Control group was injected with dextran solution without EVs. After stereomicroscope evaluation of proper EVs injection, embryos were transferred to fresh E3 medium for recovery for 6 h at 28.5°C. 54 hpf, prior to cells injection, 1x10^6^ SK-N-DZ, IMR32 cells and MSCs were labeled with Vybrant DiI Cell-Labeling Solution according to the manufacturer’s instructions. Cells were resuspended in PBS 1X and kept on ice until transplantation. Embryos were anesthetized with 200 mg/L Tricaine and placed on the EmbryoAligner containing a solution of 1.5% agarose in E3 medium. Cells suspension was loaded into borosilicate glass capillary needles (1.0 mm in diameter, World Precision Instruments, Sarasota, FL, USA) and injected into the Duct of Cuvier of the embryos using a pneumatic microinjector (World Precision Instruments) under the fluorescent stereomicroscope. By setting pressure and duration, we injected approximately 100 cells per embryo, as determined by standard cell counting within droplets. Injected embryos were transferred to fresh E3 medium for recovery for 1 h at 28.5°C. The presence of fluorescent tumor cells at the injection site and in the bloodstream was evaluated using the fluorescent stereomicroscope. Images after cells injection were captured by confocal microscope at 10X magnification. Embryos were then incubated at 34°C for the rest of the experiment. Caudal plexus images were captured by confocal microscopy at 72 hpf (10X magnification, Z-stack) and tumor cells area in the caudal plexus was analyzed using ImageJ software.

### Statistical analysis

Graphs and statistical analyses were performed using GraphPad Prism software. All data in graphs are from at least three independent experiments±SEM. Statistical significance was determined by unpaired Student’s t-test or two-way ANOVA. Asterisks indicate a significant difference between the treated and the control groups, unless otherwise specified. * p < 0.05, ** p < 0.01, *** p < 0.001, **** p < 0.0001.

## Results

### Characterization of EVs isolated from SK-N-DZ and IMR32 cells cultured at different oxygen conditions

**Fig 1:**
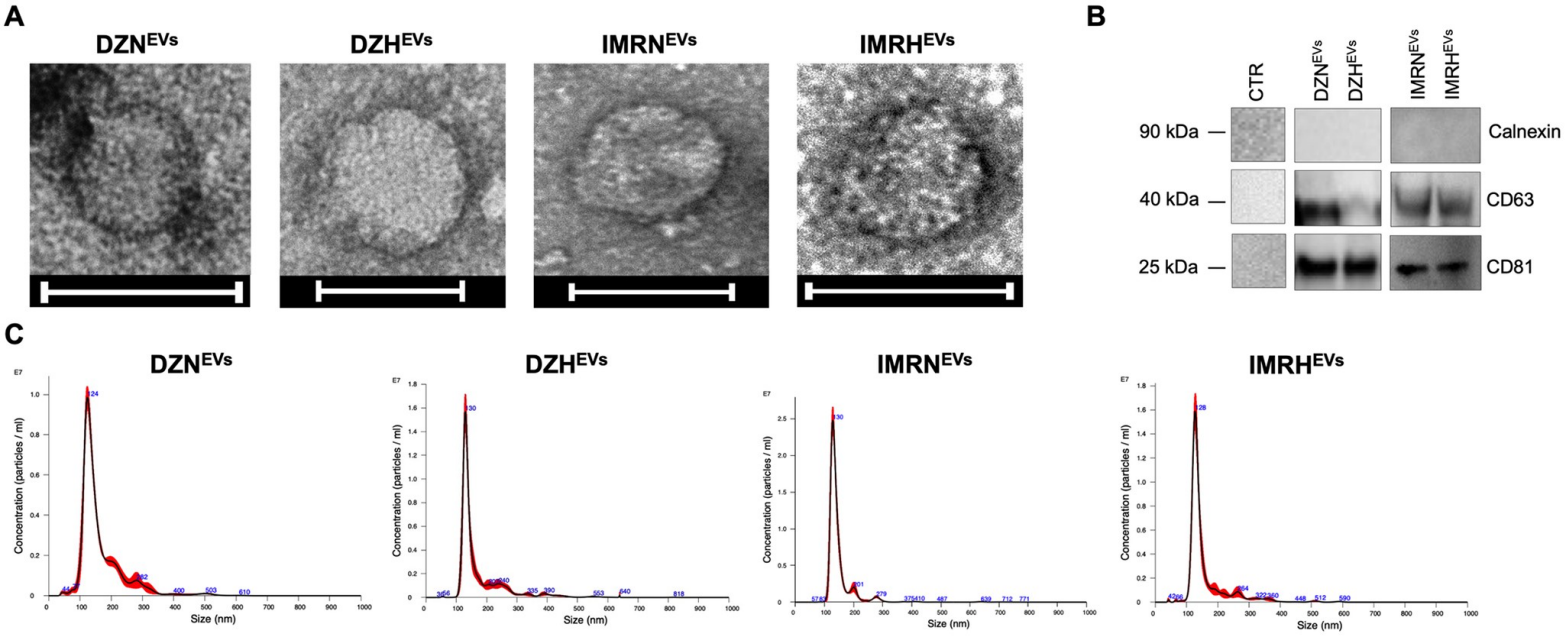
Characterization of EVs isolated from NB cells. **A.** Representative images of EVs morphology taken by TEM. Scale bar: 100 nm. **B.** Validation of EVs markers CD63, CD81, and negative marker Calnexin expression by western blot analysis. Panels represent different blots. Fresh cell culture medium was considered as CTR. **C.** Size distribution profile of EVs analyzed by NTA.

All experiments were performed using two *MYCN*-amplified NB cell lines: SK-N-DZ derived from a metastatic site and IMR-32 derived from a primary tumor. NB cells were cultured at 20% O_2_ (normoxia) or 1.5% O_2_ (hypoxia) before conditioned media (CM) collection and EVs isolation. The morphology of EVs isolated from normoxic (DZN^EVs^ IMRN^EVs^) and hypoxic (DZH^EVs^, IMRH^EVs^) conditions, was analyzed by transmission electron microscopy (TEM). Isolated EVs had a homogeneous size, consistent for both cell lines and unaffected by the oxygen tension at which the parent NB cells were cultured (Fig 1A). The expression of the characteristic EVs markers CD63, CD81 and the absence of negative marker Calnexin was verified by western blotting (Fig 1B). Furthermore, Nanoparticle Tracking Analysis (NTA) resulted in a conserved distribution profile of EVs with an average size of ~100 nm for all conditions (Fig 1C). These data indicate that EVs were successfully isolated from the CM, regardless of the oxygenation condition of the parent cells.

### Zebrafish EmbryoAligner mold design and application

**Fig 2:**
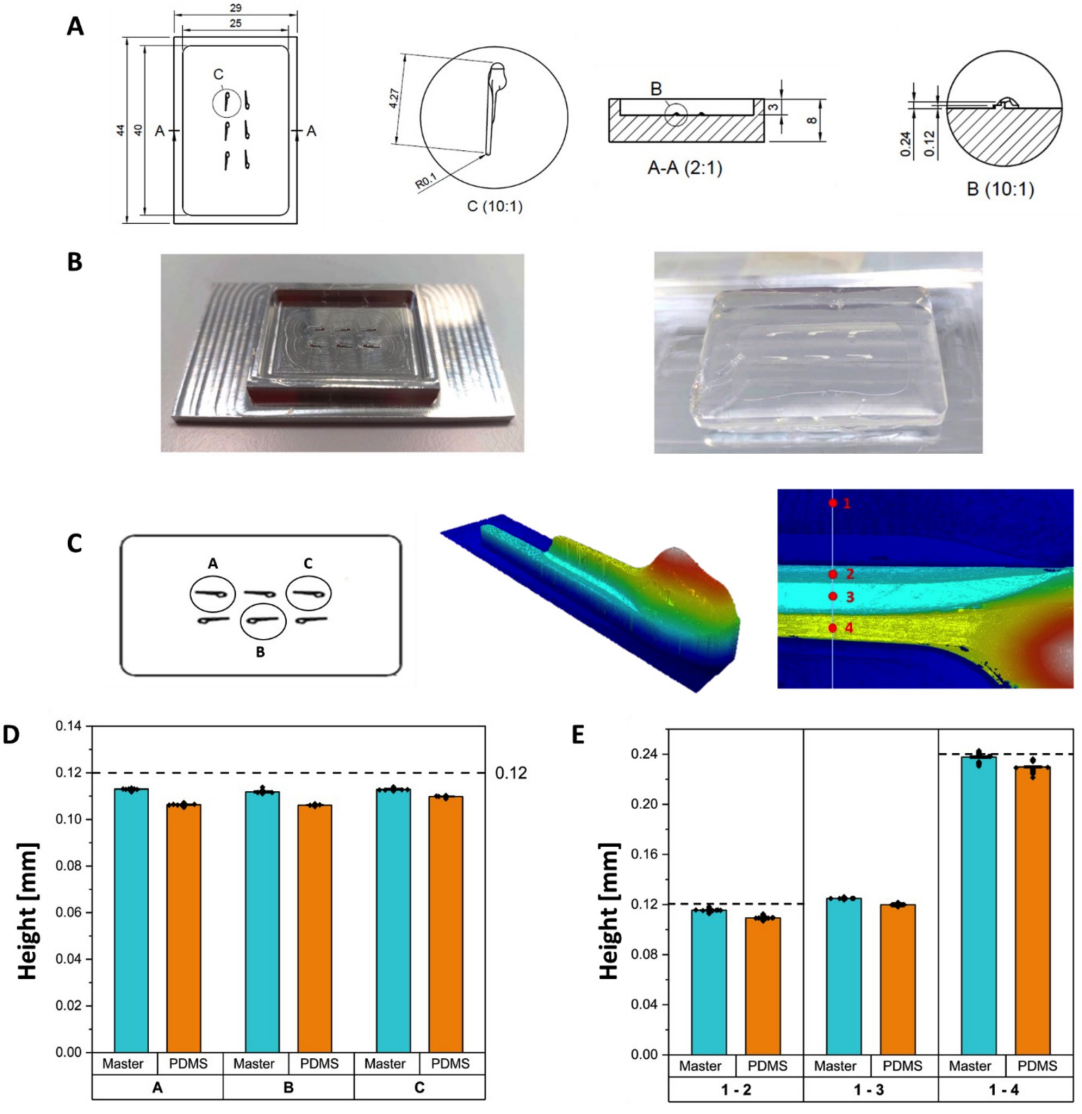
EmbryoAligner design, production, and characterization. **A.** 3D-CAD design of the master; dimensions in mm. **B.** Finished EmbryoAligner aluminum mold and PDMS replica. **C.** Selected areas of interest for the metrological analyses. **D.** Metrological characterization of the tail height. Comparisons between master mold and PDMS replicas measured in the highlighted areas of interest A-C. CAD reference value was 0.12 mm. **E.** Metrological characterization of the body height. Comparisons between master mold and PDMS replicas measured in the highlighted areas of interest 1–4. CAD reference values were 0.12 mm for the region 1–2 and 0.24 mm for 1–4. **D.** and **E.** are box mean bars with square error.

The microinjection of zebrafish embryos is a critical technique employed in numerous models. To simplify and streamline key pre- and during-injection steps such as embryo manipulation for alignment and securing in place, we designed and produced a customized tool (Fig 2). Panels A-B show the final design, the aluminum mold, and an agarose replica of the EmbryoAligner. Each replica housed up to 6 fish, aligned and kept in place in the respective slots to perform injections at different points. Given our experimental needs, the mold was designed replicating the size of embryos at 48 hpf. Panels C-E report the results of the profilometer analysis. For the aluminum master, the mean height of the tail was 0.113 ± 0.003 mm, slightly below the nominal CAD value set at 0.12 mm. The body measured 0.237 ± 0.003 mm, with a CAD value of 0.24 mm. These small deviations can be correlated to the default machine error and are within the expected tolerances. Given the poor optical properties of agarose, measures on replicas were performed using PDMS. Here, the height for the tail and body were 0.107 ± 0.0017 and 0.229 ± 0.003 mm, respectively. Thanks to the EmbryoAligner device, embryos were quickly aligned by gently pushing them with a pipette tip. After the injection, embryos were removed from the agarose device with a Pasteur pipette and transferred to a petri dish with E3 medium. The mold that we created is an easy and quick method that greatly reduces embryo manipulation while improving injection accuracy. The device is reusable, and several replicas can be created for each experiment.

### EVs visualization in the blood flow

Circulating EVs play crucial roles as mediators of cell communication between different tissues and are involved in a wide range of cellular processes [23]. *In vivo* distribution and kinetics of EVs shedding and internalization can be accurately assessed using non-invasive imaging techniques, for a more comprehensive understanding of their therapeutic effects *in vivo* [24]. The transgenic line *Tg(gata1*:*dsRed)* has dsRed-positive primitive erythroid cells, enabling visualization of labeled EVs in the blood flow. Briefly, we labeled normoxic EVs using the Vybrant DiO Cell-Labeling Solution, injected them into the zebrafish embryo at 48 hpf and immediately evaluated their presence *in vivo*. EVs could be seen as clusters in the blood circulation, with the majority homing within 30 minutes from injection (S1 Video). EVs stopped mainly in the CHT, a highly vascularized region of the zebrafish embryo [25]. These observations suggest that *in vivo* interactions between cells and EVs are a rapid process, supporting the choice of relatively short timepoints through our investigation.

### EVs internalization by endothelial cells

**Fig 3:**
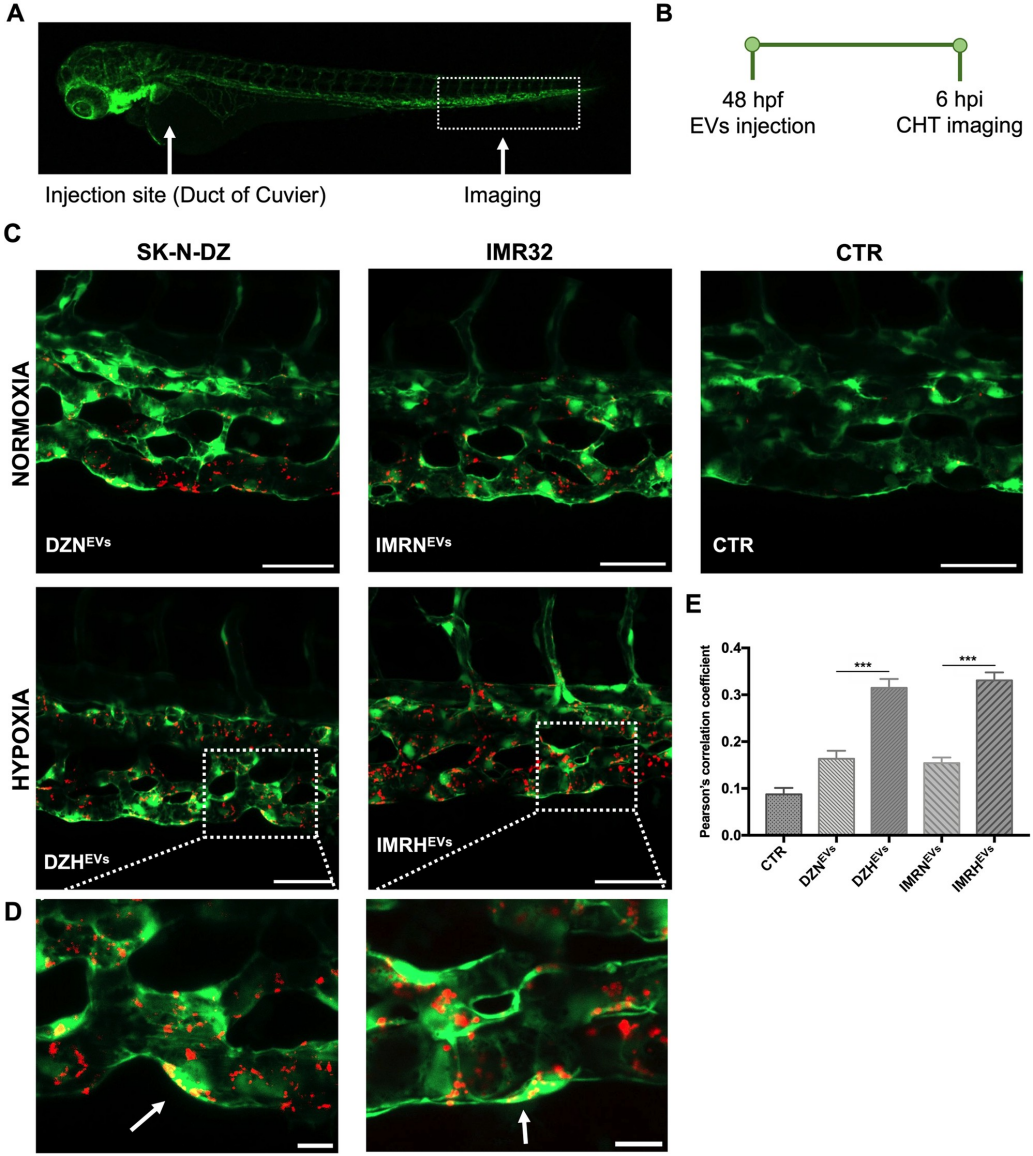
Internalization of NB-derived EVs by endothelial cells. **A-B.** Injection site, imaging site, and timeline for the transgenic line *Tg(fli1*:*EGFP)* injected with labeled NB-derived EVs (red) at 48 hpf. The CHT was imaged after 6 hours to evaluate internalization by endothelial cells (green). **C.** Representative 6 hpi CHT images of internalized EVs derived from each cell line and oxygenation condition as indicated. Scale bars 50 μm. **D.** Higher magnification images in selected areas. White arrows indicate overlapping areas (yellow) of labeled EVs (red) internalized by endothelial cells (green). Scale bars 10 μm. **E.** Evaluation of internalization of labeled EVs by endothelial cells via ImageJ software. n = 88 from at least 3 independent experiments. *** p < 0.001.

The aggressiveness and propensity to metastasize of a solid tumor like NB also depend on changes in the tumor niche, including angiogenesis. To address whether EVs affected angiogenesis *in vitro* we analyzed changes in the ability of endothelial cells to form capillary-like structures using a tube formation assay on Matrigel (S1 Fig). Our findings indicate that EVs from hypoxic conditions significantly increased the number of nodes and total tube length in network structures compared to untreated controls. To assess the ability of EVs to be internalized by recipient cells and study their effect on endothelial cells *in vivo*, we used the *Tg(fli1*:*EGFP)* transgenic line expressing enhanced green fluorescent protein (EGFP) in endothelial cells (Fig 3). To evaluate whether different oxygen tensions could affect the internalization of EVs by endothelial cells, we injected 23 nL of labeled NB-derived normoxic and hypoxic EVs into *Tg(fli1*:*EGFP)* embryos at 48 hpf (Fig 3A and 3B). EVs were rapidly internalized by endothelial cells 30 minutes post injection (S2 Video). The CHT (Fig 3C and 3D) were then imaged at 6 hpi, proving that EVs derived from NB cells cultured in hypoxic conditions were internalized more when compared with their normoxic counterparts. Quantifications of the correlation coefficient gave values of 0.316±0.02 and 0.175±0.02 for DZH^EVs^ and DZN^EVs^, respectively, and 0.323±0.02 and 0.150±0.02 for IMRH^EVs^ and IMRN^EVs^ (Fig 3E). This suggests that a greater internalization of EVs leads to an increased transportation of their cargo into recipient cells, thereby impacting them.

### Hypoxic NB-derived EVs promote angiogenesis

**Fig 4:**
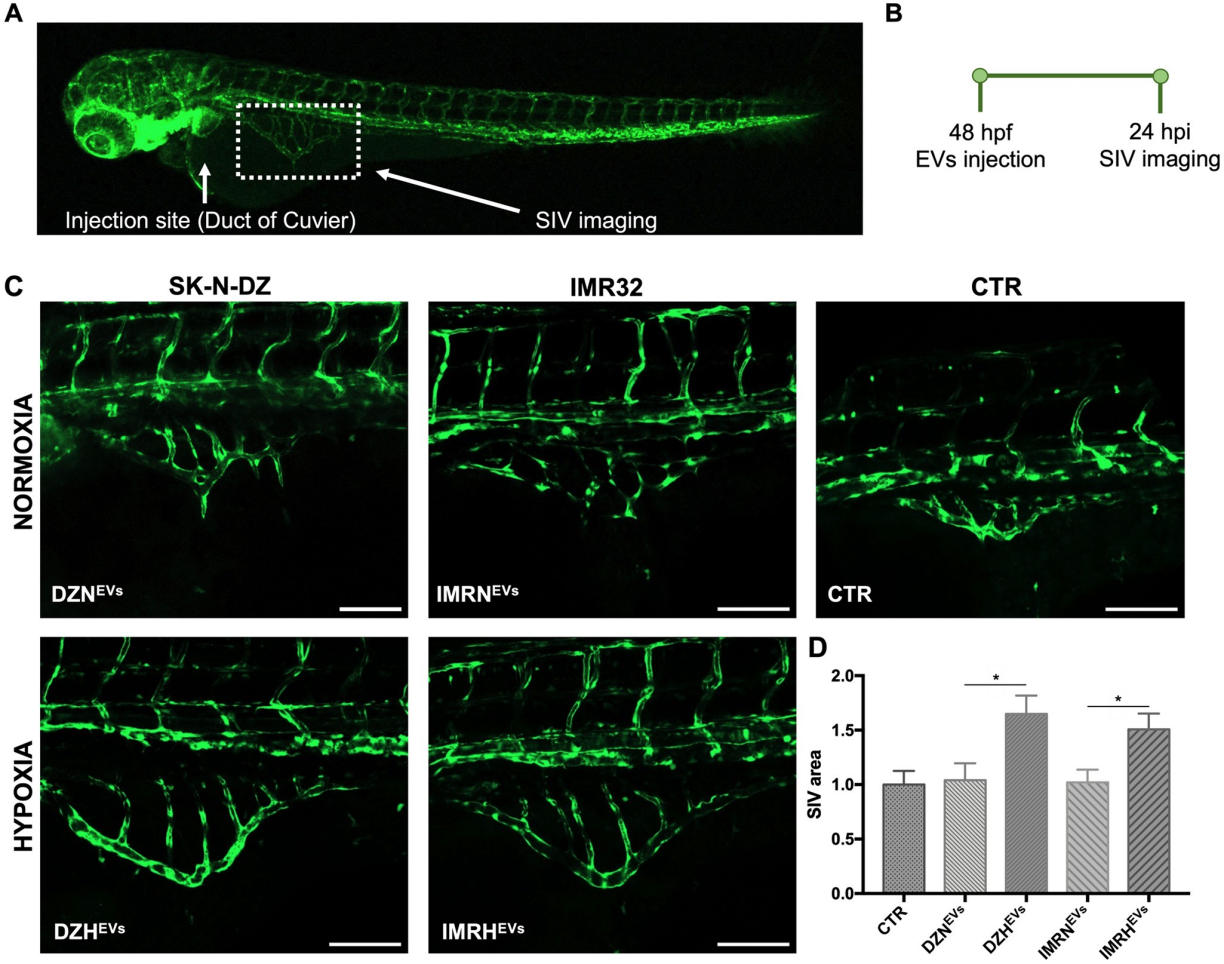
Effects of NB-derived EVs on angiogenesis. **A-B.** Injection site, imaging site, and timeline for the transgenic line *Tg(fli1*:*EGFP)* injected with NB-derived EVs at 48 hpf. **C.** SIVs were imaged 24 hpi to evaluate the sprouting of vessels. SIVs areas analyzed are highlighted by white dotted lines. Scale bars 100 μm. **D.** Evaluation of SIVs area via ImageJ software. n = 137 from at least 3 independent experiments. * p < 0.05.

Angiogenesis plays an important role in tumor growth, invasion, and metastasis, and is known to be induced, among other factors, by hypoxia [26]. SIVs sprout from the Duct of Cuvier and connect to the posterior cardinal vein and are used as a model to study venous angiogenesis [9, 27, 28]. To evaluate whether hypoxic EVs can stimulate angiogenesis, we measured the SIVs area of *Tg(fli1*:*EGFP)* embryos at 24 hpi (Fig 4A–4C). We found that the injection of DZH^EVs^ induced 60% more sprouting of vessels in the SIVs compared to the embryos injected with DZN^EVs^. Similarly, the injection of IMRH^EVs^ induced 50% more sprouting compared to the embryos injected with IMRN^EVs^ (Fig 4D). These results demonstrate that hypoxic NB EVs are indeed strong effectors of angiogenesis *in vivo*. Furthermore, as shown in S3A and S3B Fig, the injection of EVs isolated from mesenchymal stem cells (MSCs), cultured under normoxic and hypoxic conditions, revealed that these EVs had no significant effect on angiogenesis compared to the control group. This indicates that non-tumor EVs did not stimulate angiogenesis in zebrafish embryos.

### EVs are internalized by macrophages and can affect their proliferation

**Fig 5:**
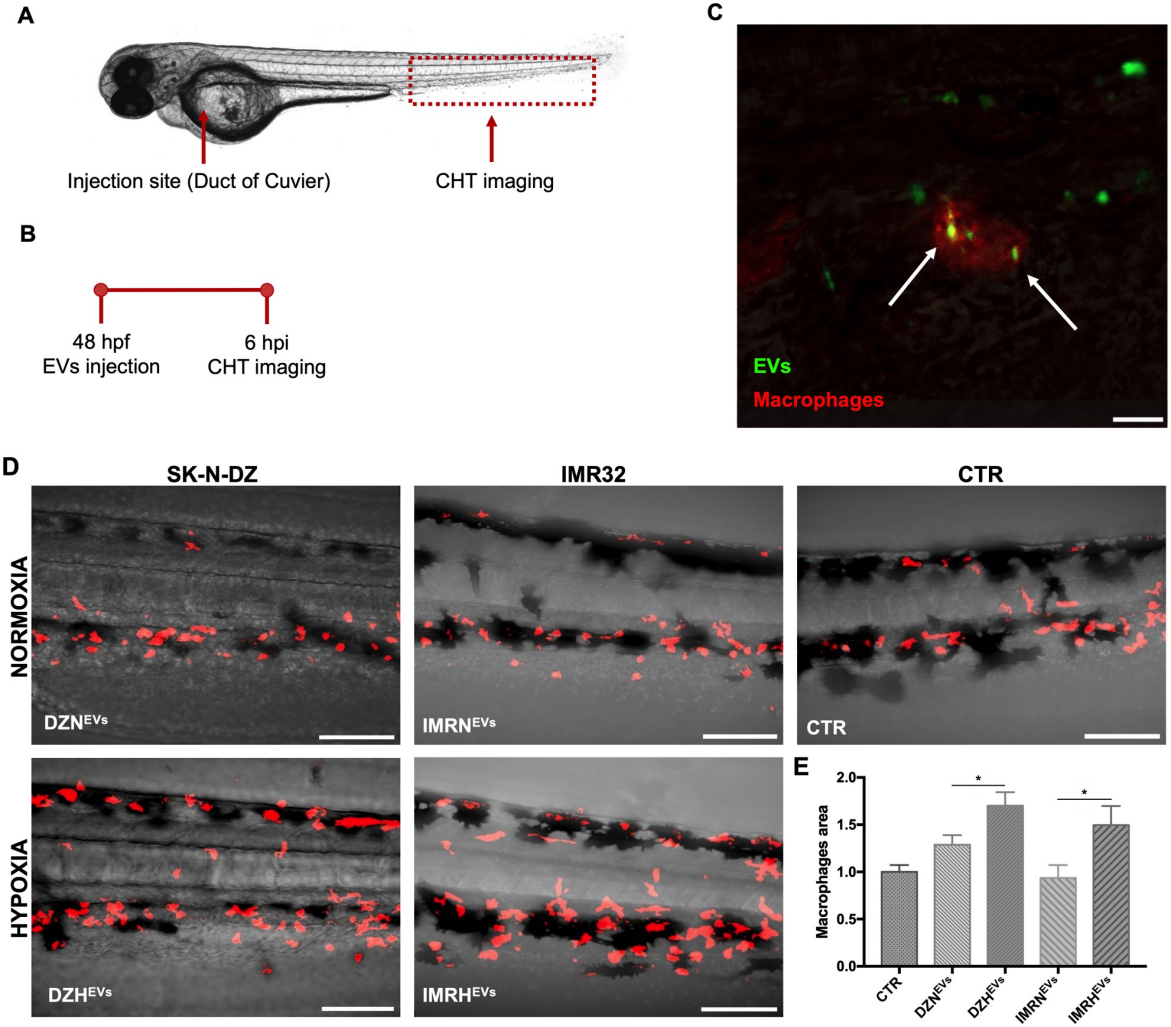
Hypoxic NB-derived EVs promote macrophages mobilization. **A-B.** Injection site, imaging site, and timeline for the transgenic line *Tg(mpeg1*:*mCherry)* injected with NB-derived EVs at 48 hpf. **C.** Representative image of labeled EVs (green, white arrows) internalized by macrophages (red). Scale bar 20 μm. **D.** Macrophages mobilization in the CHT 6 hpi. Scale bars 100 μm. **E.** Evaluation of macrophages area via ImageJ software. n = 125 from at least 2 independent experiments. * p < 0.05.

EVs are also responsible for the modulation of inflammation in disease progression [29]. EVs derived from cancer cells have the potential to significantly alter the phenotypes and functions of macrophages, thereby promoting tumor development [30]. To investigate the effects of EVs on macrophages, we injected the transgenic line *Tg(mpeg1*:*mCherry)* with 23 nL of NB-derived EVs at 48 hpf (Fig 5A and 5B). This transgenic line specifically labels macrophages, enabling the visualization of their behavior *in vivo* [18]. Patrolling macrophages successfully internalized tumor EVs (Fig 5C, S3 Video). To evaluate the effects of EVs on macrophages proliferation/mobilization, we analyzed the area covered by macrophages in the zebrafish embryo at 6 hpi (Fig 5D). Compared to normoxic DZN^EVs^ and IMRN^EVs^ (areas of 1.287±0.1, and 0.936±0.1 respectively), we demonstrated that hypoxic DZH^EVs^ and IMRH^EVs^ (1.701±0.14 1.496±0.2, respectively) significantly promoted macrophages proliferation (Fig 5E). Although the association between the number of macrophages and their mobilization in tumor progression is still uncertain, these data indicate that hypoxic EVs isolated from NB SK-N-DZ and IMR32 cell lines target macrophages by inducing their mobilization.

### Hypoxic EVs influence the expression of mmp9 and cxcl8b in the CHT

**Fig 6:**
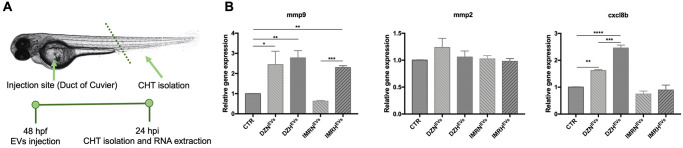
Hypoxic EVs influence the expression of mmp9 and cxcl8b in the CHT. **A.** Injection site, analysis site, and timeline for Casper embryos injected with NB-derived EVs. **B.** Relative mRNA expression for mmp9, mmp2 and cxcl8b measured by qPCR in CHT tissues 24 hours after EVs injection. Data are represented as 2^-ΔΔCt^, β-Actin was used as housekeeping gene. n = 450 from at least 3 independent experiments. * p < 0.05, ** p < 0.01, *** p < 0.001, **** p < 0.0001.

Like the phenotype of the mammalian bone marrow, the CHT is the first homing and expansion site for hematopoietic stem cells in the zebrafish embryo [31]. Zebrafish and mammals also share similarities in key cytokines and chemokines such as CXCL12, necessary for the hematopoietic stem and progenitor cells guidance through the CXCL2–CXCR4 signaling axis [32, 33]. To mimic metastatic colonization in the CHT and investigate the effects of NB-derived EVs on the CHT, we injected the EVs in the blood circulation of *Casper* zebrafish embryos at 48 hpf, isolated CHT tissues at 24 hpi (Fig 6A), and analyzed the expression of the inflammatory chemokine *cxcl8b* [34], and the matrix metalloproteinase-9 *(mmp-9)* and *mmp-2*, which correlate with neuroblastoma metastasis and progression (Fig 6B) [35–38]. We found that the expression of *mmp-9* was significantly increased in the embryos injected with DZN^EVs^ and DZH^EV^ (2.4- and 2.8-fold, respectively) compared to the control group. The expression of *mmp-9* increased 2.3-fold in the group injected with IMRH^EVs^ and decreased 1.6 fold in the IMRN^EVs^-injected group compared to the control group. No significant changes were measured in *mmp-2* expression between normoxic and hypoxic-EVs from both cell lines. Finally, we demonstrated that the expression of *cxcl8b* significantly increased in the embryos injected with DZN^EVs^ and DZH^EVs^ (1.6 and 2.4-fold, respectively) compared to the control group. The expression of *cxcl8b* did not differ in the embryos injected with IMR32-derived EVs. These findings indicate that EVs indeed influence the expression of genes involved in metastatic progression, with different effects depending on the parental cells of origin. EVs derived from the metastatic SK-N-DZ cells, are more potent than those from IMR32, a primary tumor cell line.

### Hypoxic EVs promote NB cells proliferation in the CHT

**Fig 7:**
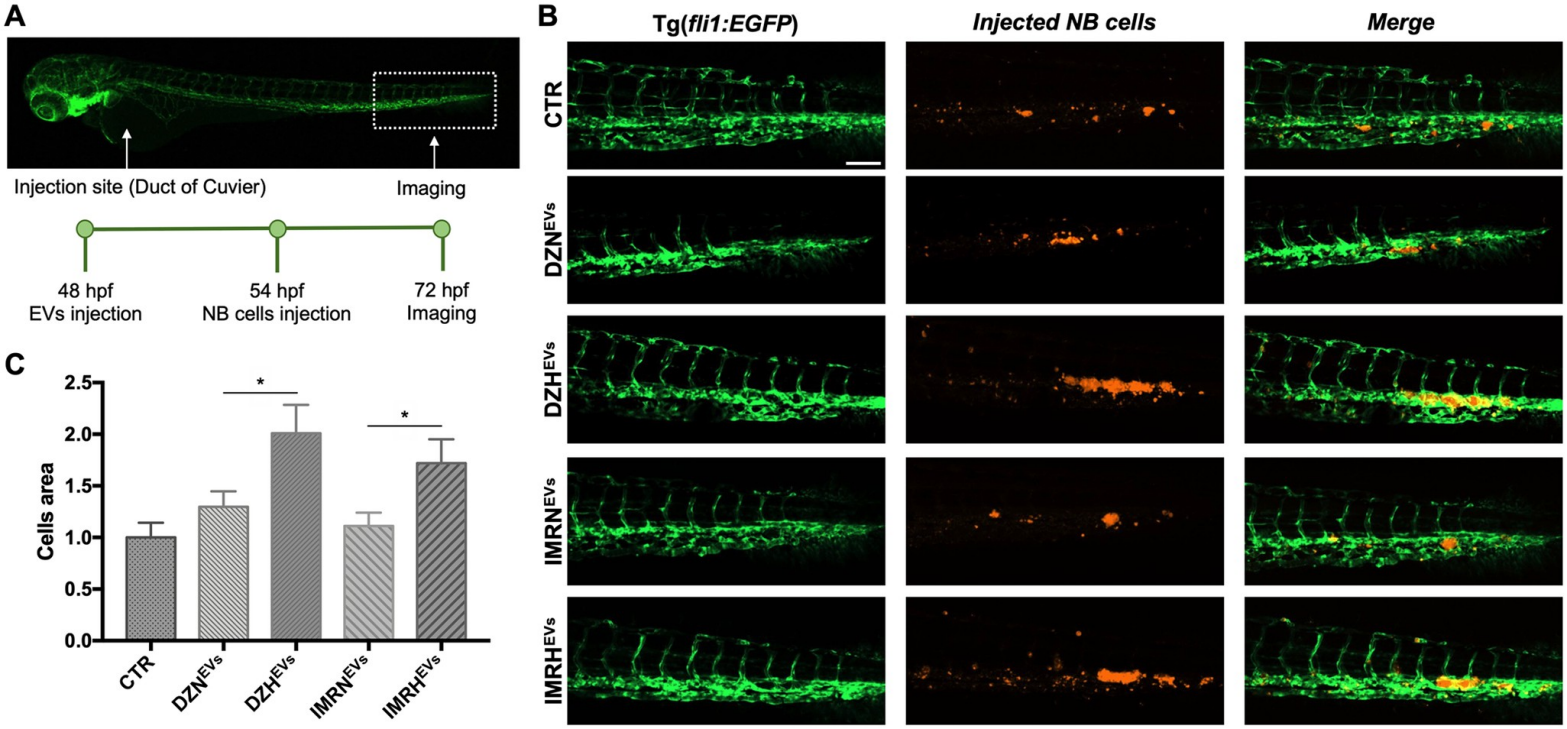
Hypoxic EVs promote the proliferation of NB cells in the CHT. **A.** Injection site, analysis site, and timeline for *Tg(fli1*:*EGFP)* embryos injected with NB-derived EVs and NB cells. Embryos were injected with EVs isolated from SK-N-DZ or IMR32 cells cultured in normoxic or hypoxic conditions, and subsequently injected with SK-N-DZ and IMR32 cells, respectively. **B.** Representative CHT images 24 hpi of EVs and 18 hpi of SK-N-DZ cells (orange). Scale bar 100 μm. **C**. Representative CHT images 24 hpi of EVs and 18 hpi of IMR32 cells (orange). Scale bar 100 μm. **D.** Evaluation of proliferated SK-N-DZ cells area in the CHT at 72 hpf. n = 53 from at least 3 independent experiments. * p < 0.05. **E.** Evaluation of proliferated IMR32 cells area in the CHT at 72 hpf. n = 59 from at least 3 independent experiments. * p < 0.05.

Although the key role of EVs in the preparation of the pre-metastatic niche and tumor establishment has been extensively studied [39–41], it has never been investigated in NB using an *in vivo* zebrafish model. We mimicked metastatic colonization by a two-step injection in *Tg(fli1*:*EGFP)* embryos 48 hpf (Fig 7A): first with NB-derived EVs, and 6 hours later with NB cells (S2 Fig). NB cells proliferation in the CHT was evaluated at 24 hpi (Fig 7B). DZH^EVs^-injected embryos had a higher NB cell area compared to their normoxic counterpart (2.01±0.2 and 1.3±0.2, respectively). A similar trend was measured for IMR^EVs^-injected embryos (1.72±0.2 and 1.11±0.13 for hypoxic and normoxic EVs, respectively) (Fig 7C). These data suggest that hypoxia induces NB cells to shed EVs that are more potent in preconditioning and targeting the potential metastatic site and modifying the CHT microenvironment by promoting NB cells proliferation. Furthermore, we proved that this effect is specific to NB cells and does not occur in non-tumor cells like MSCs (please see S3C–S3E Fig). Similarly, MSCs-derived EVs had no effect on tumor cell proliferation (S3G and S3H Fig). These findings, along with the results reported above, demonstrate that the homing and proliferation of tumor cells in the CHT is mediated by tumor-derived EVs, rather than by simple accumulation caused by blood circulation.

## Discussion and conclusions

Cancer cell derived EVs emerged as potential mediators of metastatic spread and of the pre-metastatic niche formation [42], while hypoxia, a decline of tissue oxygen level, is a known hallmark of cancer favoring tumor growth, angiogenesis, and metastasis [43]. In our recent study, we demonstrated the ability of hypoxic EVs to increase the aggressiveness of NB cells *in vitro* [44]. Here, we studied the role of hypoxic and normoxic EVs in NB metastatic dissemination in an *in vivo* zebrafish (*Danio Rerio)* model using specific transgenic lines [45, 46]. We first characterized purified EVs released by two MYCN amplified NB cell lines cultured in normoxic and hypoxic conditions, proving their similarities in morphology, size, and surface markers independently on the culture conditions of the parent cells. Before moving to the *in vivo* experiments, we designed and fabricated a customized device to minimize manipulation of the zebrafish embryos and increase repeatability of our experiments. Each device could accommodate and correctly position 6 embryos, greatly facilitating and reducing errors during injections into the Duct of Cuvier. This site was chosen as it ensures a rapid and complete dissemination of exogenous components throughout the blood circulation [47].

Injected fluorescently labeled-EVs preferentially home in the CHT of *Tg(gata1*:*dsRed)* embryos. The CHT is a highly vascularized region in the zebrafish tail [25] sharing similarities with the cytoarchitecture of the mammalian bone marrow [31], the main metastatic site in NB. Furthermore, *Tg(fli1*:*EGFP)* embryos expressing endothelial cells in green, allowed visualization of EVs in the blood circulation proving the higher internalization by endothelial cells of EVs derived from hypoxic NB cells compared to their normoxic counterparts. These findings confirm the EVs-mediated preferential targeting of the bone marrow by NB, and a potent role for hypoxia in facilitating EVs uptake and cargo delivery by target cells. We then proved that hypoxic EVs induced an increase of sprout areas in the SIVs of transgenic zebrafish embryos [27], further supporting their role in promoting tumor spread by favoring neoangiogenesis [48, 49]. This finding is consistent with several reports on the higher angiogenic capacity of hypoxic EVs in various cancers, supporting the increased oxygen and nutrients demand in growing tumors [50, 51]. The complex chain of events dictating tumor metastatic spread also involves the immune compartment, with macrophages promoting angiogenesis and the formation of a pre-metastatic niche through the secretion of chemokines [52] and transforming into tumor-associated macrophages (TAMs) [53, 54]. Using the transgenic line *Tg(mpeg1*:*mCherry)* we here showed that injection of hypoxic EVs effectively led to increased mobilization of macrophages compared to their normoxic counterparts, further proving the role of hypoxia and the involvement of macrophages in favoring tumor progression and angiogenesis. However, given that macrophages are remarkably heterogenous, whether it is the pro- or anti-inflammatory phenotype that increases after EVs injection remains to be investigated. Considering the preferential homing of EVs [31, 55], we collected and analyzed CHT tissues 24 hpi to measure key matrix metalloproteinases and chemokines mainly released by TAMs and known to promote angiogenesis, tumor invasion, and metastasis [56, 57]. The expression of *mmp-9* increased in embryos injected with hypoxic EVs, further evidence of the role of hypoxia on angiogenesis and macrophage function. No significant differences emerged for *mmp-2*, in line with reports where its increased expression in NB patients was observed at advanced stages and in those who died from progressive disease [37]. The slight variation we observed at our 24 hpi timepoint leads to the hypothesis that analyses at longer timepoints, with both normoxic and hypoxic EVs, might result in higher differences for both *mmp-9 and mmp-2* expression. C*xcl8b* expression increased only after injection of EVs derived from the metastatic SK-N-DZ cells, supporting the hypothesis that EVs released by a metastatic line have a greater effect compared to the EVs released by a primary tumor cell line.

Last, we mimicked the steps of NB metastatic niche establishment with a double injection of EVs at 48 hpf, followed by NB cells 6 hours later. Timings were determined based on the effects of EVs on angiogenesis and macrophages mobilization which we analyzed at 6 hpi and the fact that the duct of Cuvier physiologically disappears at later timepoints. Hypoxic EVs were again more potent drivers of NB aggressiveness by increasing NB cells proliferation in the CHT 24 hours post injections compared to their normoxic counterparts.

Our findings suggest that EVs derived from NB cells play a crucial part in NB spread and the formation of pre-metastatic niches with hypoxia emerging as a potent factor influencing the function of EVs by inducing angiogenesis, macrophages recruitment, and affecting the expression of *mmp-9* and *cxcl8b*. Furthermore, mimicking the steps of NB metastatic niche establishment confirmed that the pre-conditioning of the CHT with hypoxic EVs promotes the proliferation of NB cells. Overall, we also highlight the ability of the zebrafish to serve as potent tool to study the *in vivo* interaction between EVs and cells, thanks to the combination of transgenic lines and high-resolution *in vivo* imaging. Although longer time points are necessary to fully follow the complex temporal sequence of events leading to metastatic spread, we believe that our study gives proof of the potency of this model. By enabling to better understand the key pathways at play and the underlying molecular mechanisms, we are confident that further studies will lead to better cures and targeted therapies for NB and beyond.

## Supporting information

S1 Table List of all primers. (TIFF)

S1 Video DiO-labeled NB-derived EVs in the blood stream. Representative CHT video of labeled EVs (green) injected at 48 hpf in the Duct of Cuvier of Tg(gata1:dsRed) zebrafish embryos expressing red blood cells in red. Magnification 25X. Scale bar 10 μm.
(AVI)

S2 Video DiI-labeled NB-derived EVs are internalized by endothelial cells. ISV (intersegmental vessel) representative video of labeled EVs (red) injected in the Duct of Cuvier of Tg(fli1:EGFP) zebrafish embryos at 48 hpf internalized by endothelial cells (in green). Magnification 25X. Scale bar 5 μm.
(AVI)

S3 Video DiO-labeled NB-derived EVs are internalized by macrophages. Representative video of labeled EVs (green) injected at 48 hpf in the Duct of Cuvier of Tg(mpeg1:mCherry) zebrafish embryos internalized by macrophages (in red). Magnification 25X. Scale bar 20 μm.
(MP4)

S1 Fig Tube forming assay. The angiogenic property was assessed by measuring the total branching point and tube length from five random microscopic fields using Python Angiogenesis analyzer.
(TIFF)

S2 Fig NB cells in the CHT after injection. Acquisition at 1 hpi of Tg(fli1:EGFP) embryos injected at 54 hpf with NB cells. Magnification 10X. Scale bar 100 μm.
(TIFF)

S3 Fig Effects of MSCs-derived EVs on angiogenesis and on NB cells proliferation. **A.** Tg(fli1:EGFP) embryos were injected with MSCs-derived EVs at 48 hpf. SIVs were imaged 24 hpi to evaluate the sprouting of vessels. SIVs areas analyzed are highlighted by white dotted lines. Scale bars 100 μm. **B.** Evaluation of SIVs area via ImageJ software. n = 32. **C.** Timeline for Tg(fli1:EGFP) embryos injected with NB-derived EVs and MSCs. Embryos were injected with EVs isolated from SK-N-DZ cells, and subsequently injected with MSCs. **D.** Representative CHT images 24 hpi of EVs and 18 hpi of MSCs cells (orange). Scale bar 100 μm. **E.** Evaluation of proliferated MSCs area in the CHT at 72 hpf. n = 37. **F.** Timeline for Tg(fli1:EGFP) embryos injected with MSCs-derived EVs and NB cells. Embryos were injected with EVs isolated from MSCs, and subsequently injected with SK-N-DZ cells. **G.** Representative CHT images 24 hpi of EVs and 18 hpi of SK-N-DZ cells (orange). Scale bar 100 μm. **H.** Evaluation of proliferated SK-N-DZ area in the CHT at 72 hpf. n = 35. All variations were not statistically significant.
(TIFF)

S1 Raw images (PDF)

## Abbreviations

CHT: Caudal hematopoietic tissues
CTR: Control
DZH^EVs^: Extracellular vesicles derived from SK-N-DZ cells cultured in hypoxic conditions
DZN^EVs^: Extracellular vesicles derived from SK-N-DZ cells cultured in normoxic conditions
ECM: Extracellular matrix
EVs: Extracellular vesicles
hpf: Hours post fertilization
hpi: Hours post injection
IMRH^EVs^: Extracellular vesicles derived from IMR32 cells cultured in hypoxic conditions
IMRN^EVs^: Extracellular vesicles derived from IMR32 cells cultured in normoxic conditions
ISV: Intersegmental vessel
mmps: Matrix metalloproteinases
NB: Neuroblastoma
SIV: Sub-intestinal veins
TAMs: Tumor associated macrophages
