# Supplementary figures and images for: Neuroblastoma-derived hypoxic extracellular vesicles promote metastatic dissemination in a zebrafish model
Source: PLoS One. 2024 Dec 23;19(12):e0316103. doi: 10.1371/journal.pone.0316103 (PMC11666040; doi:10.1371/journal.pone.0316103)

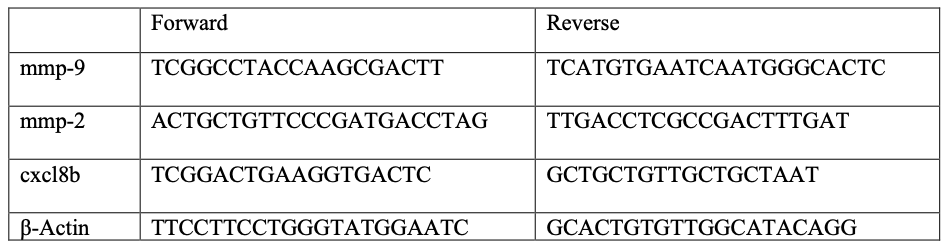

Supplement: S1 Table — (TIFF) [file pone.0316103.s001.tiff]

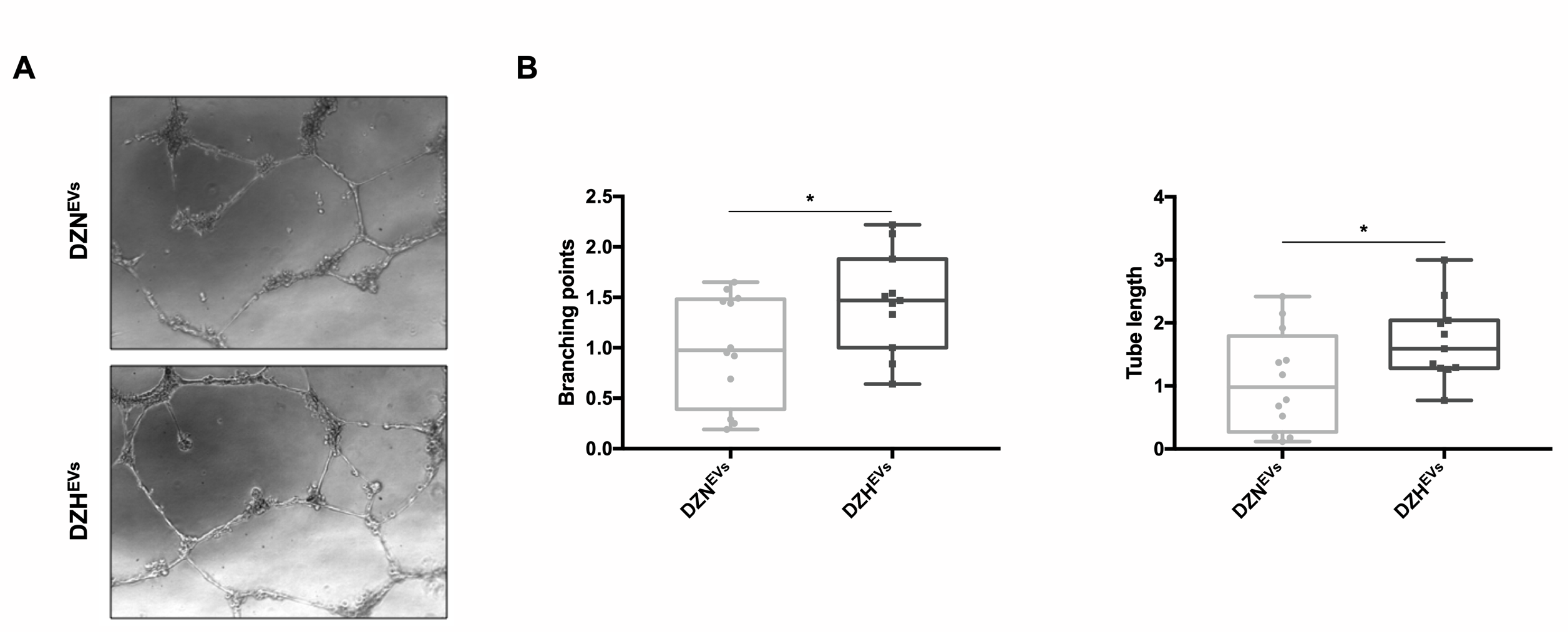

Supplement: S1 Fig — The angiogenic property was assessed by measuring the total branching point and tube length from five random microscopic fields using Python Angiogenesis analyzer. (TIFF) [file pone.0316103.s005.tiff]

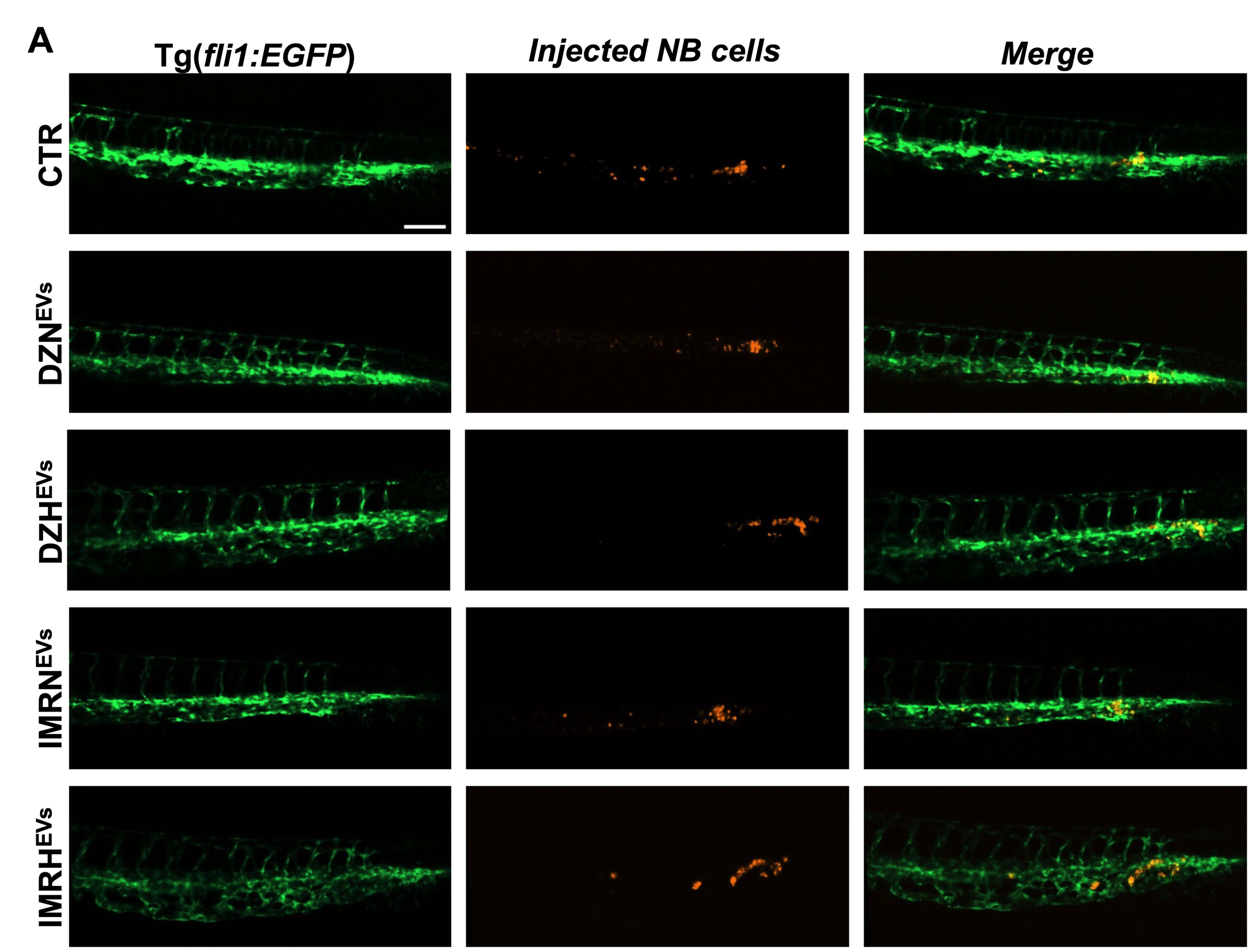

Supplement: S2 Fig — Acquisition at 1 hpi of Tg(fli1:EGFP) embryos injected at 54 hpf with NB cells. Magnification 10X. Scale bar 100 μm. (TIFF) [file pone.0316103.s006.tiff]

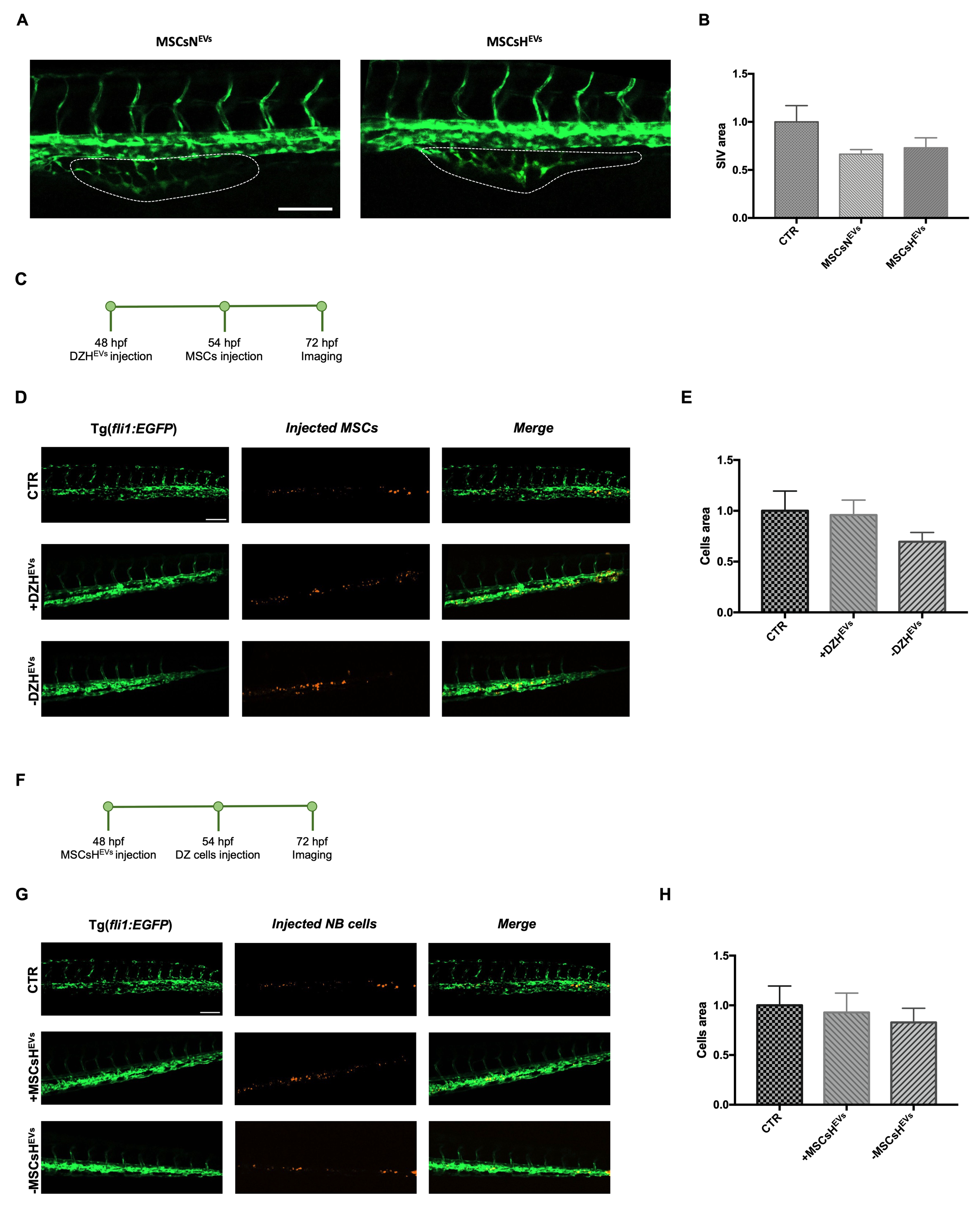

Supplement: S3 Fig — A. Tg(fli1:EGFP) embryos were injected with MSCs-derived EVs at 48 hpf. SIVs were imaged 24 hpi to evaluate the sprouting of vessels. SIVs areas analyzed are highlighted by white dotted lines. Scale bars 100 μm. B. Evaluation of SIVs area via ImageJ software. n = 32. C. Timeline for Tg(fli1:EGFP) embryos injected with NB-derived EVs and MSCs. Embryos were injected with EVs isolated from SK-N-DZ cells, and subsequently injected with MSCs. D. Representative CHT images 24 hpi of EVs and 18 hpi of MSCs cells (orange). Scale bar 100 μm. E. Evaluation of proliferated MSCs area in the CHT at 72 hpf. n = 37. F. Timeline for Tg(fli1:EGFP) embryos injected with MSCs-derived EVs and NB cells. Embryos were injected with EVs isolated from MSCs, and subsequently injected with SK-N-DZ cells. G. Representative CHT images 24 hpi of EVs and 18 hpi of SK-N-DZ cells (orange). Scale bar 100 μm. H. Evaluation of proliferated SK-N-DZ area in the CHT at 72 hpf. n = 35. All variations were not statistically significant. (TIFF) [file pone.0316103.s007.tiff]
