# Supplementary material for: Neuroblastoma-derived hypoxic extracellular vesicles promote metastatic dissemination in a zebrafish model
Source: PLoS One. 2024 Dec 23;19(12):e0316103. doi: 10.1371/journal.pone.0316103 (PMC11666040; doi:10.1371/journal.pone.0316103)
Supplement: S1 Raw images — (PDF) [file pone.0316103.s008.pdf]

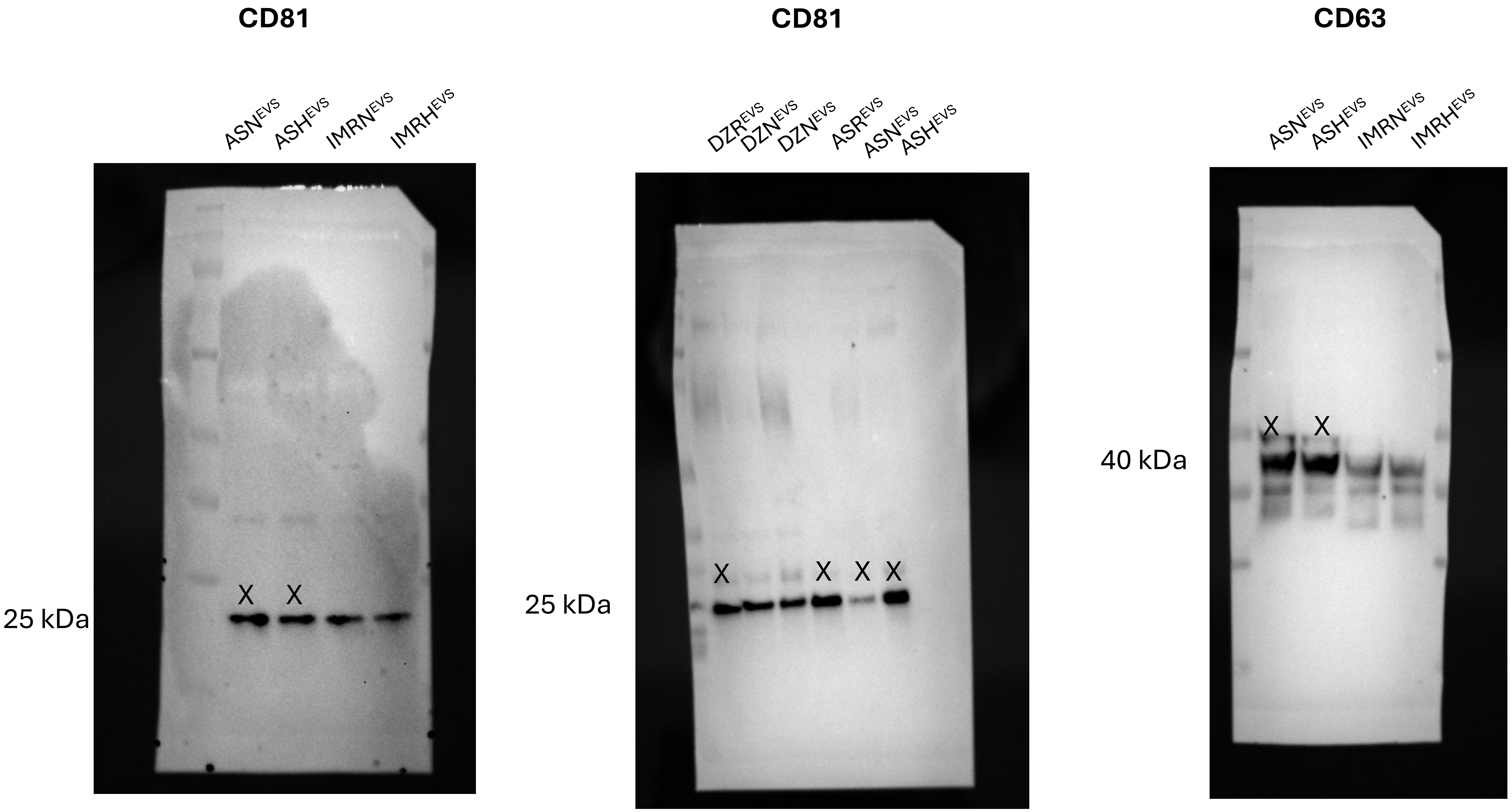
CD81
CD81
CD63
ASN^{EVS}
ASH^{EVS}
$IMRN^{EVS}$
$IMRH^{EVS}$
X
X
25 kDa
DZR^{EVS}
DZN^{EVS}
DZN^{EVS}
ASR^{EVS}
ASN^{EVS}
ASH^{EVS}
X
X
X
X
25 kDa
ASN^{EVS}
ASH^{EVS}
$IMRN^{EVS}$
$IMRH^{EVS}$
X
X
40 kDa

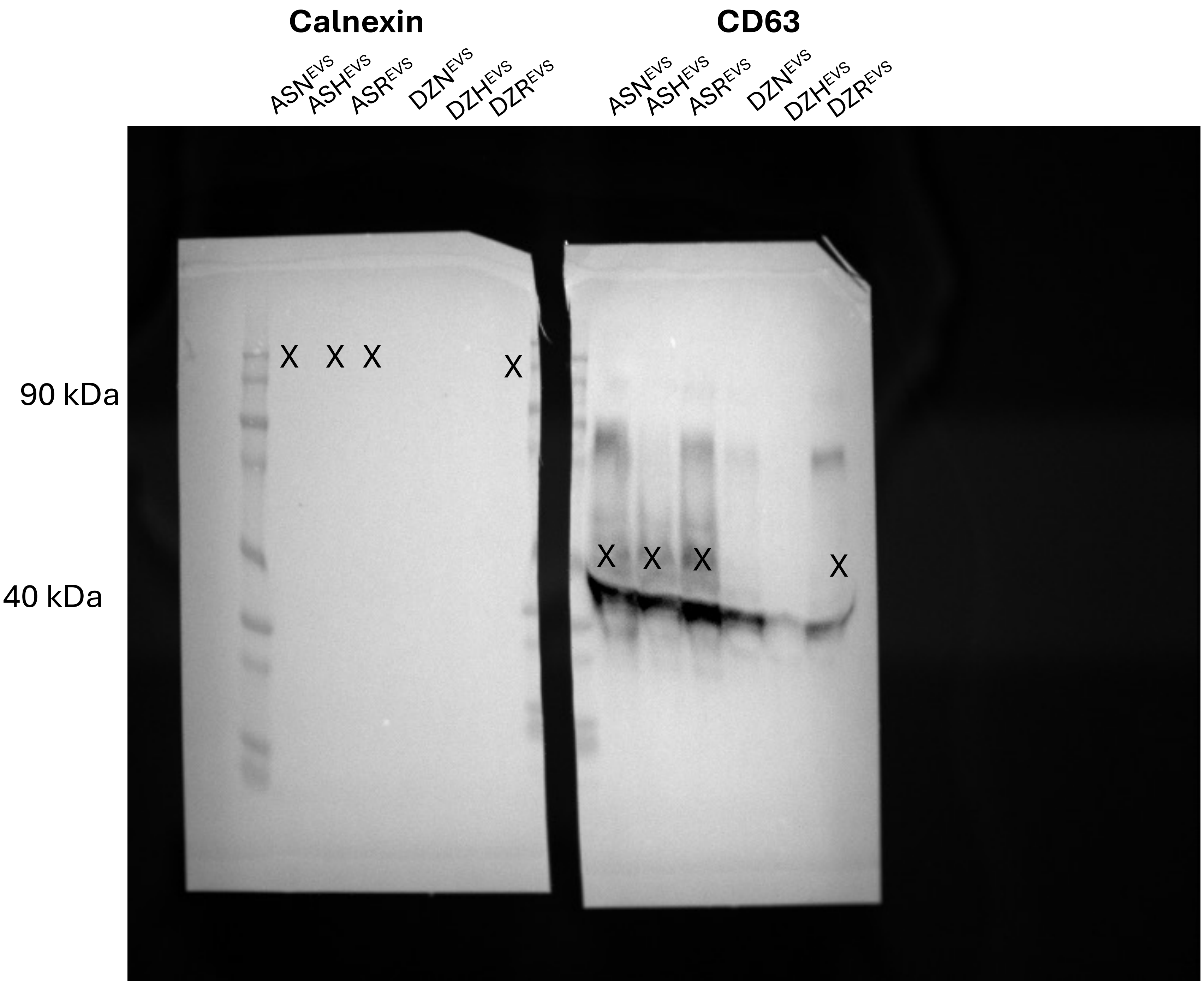

Calnexin
CD63
ASN^EVS ASH^EVS ASR^EVS DZN^EVS DZH^EVS DZR^EVS
ASN^EVS ASH^EVS ASR^EVS DZN^EVS DZH^EVS DZR^EVS
X X X X
90 kDa
X X X X
40 kDa

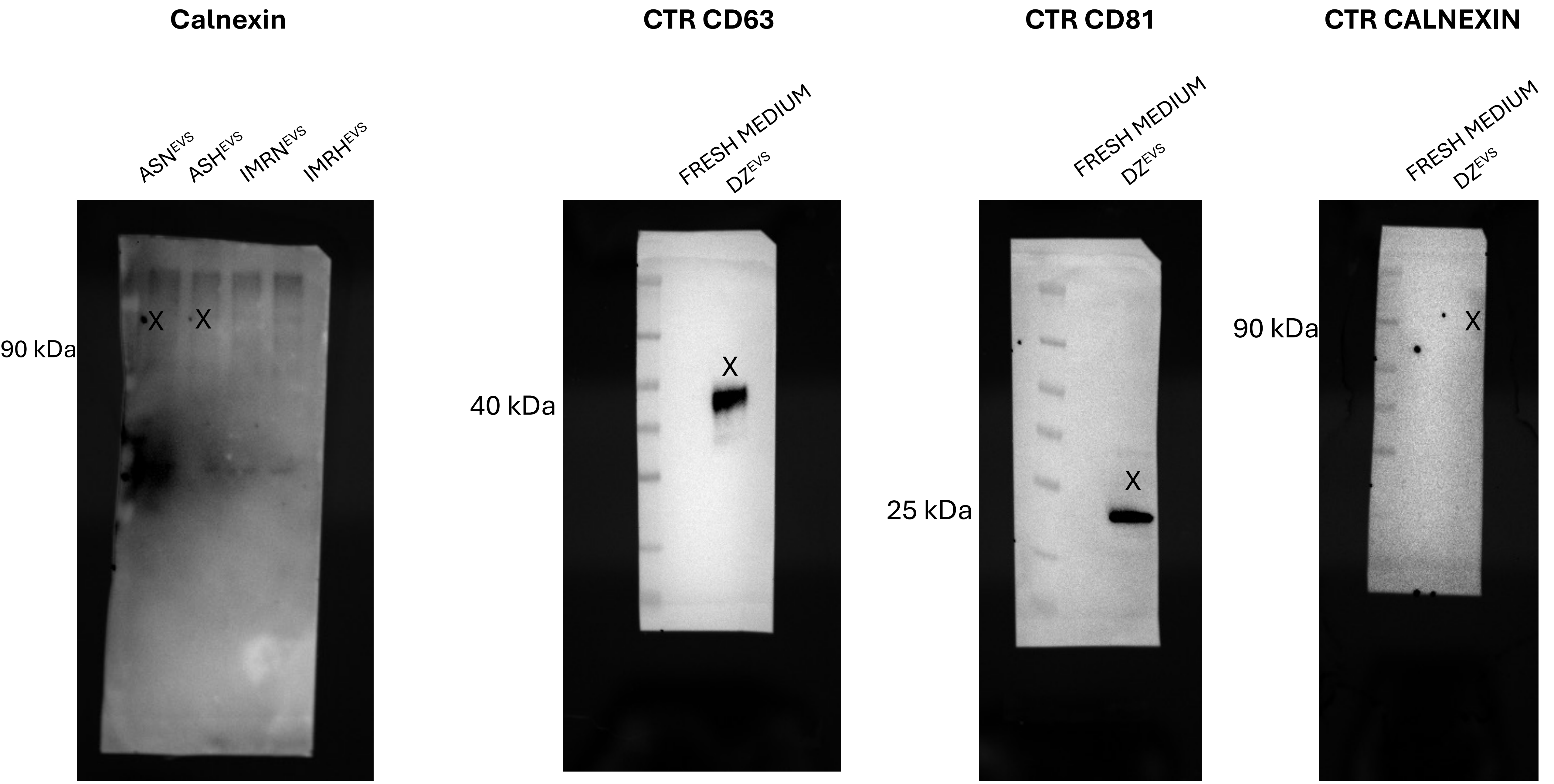
Calnexin
ASN^{EVS}
ASH^{EVS}
IMRN^{EVS}
IMRH^{EVS}
X
X
90 kDa
CTR CD63
FRESH MEDIUM
DZ^{EVS}
X
40 kDa
CTR CD81
FRESH MEDIUM
DZ^{EVS}
X
25 kDa
CTR CALNEXIN
FRESH MEDIUM
DZ^{EVS}
90 kDa
X
